# Delineating spatial cell-cell interactions in the solid tumour microenvironment through the lens of highly multiplexed imaging

**DOI:** 10.3389/fimmu.2023.1275890

**Published:** 2023-10-23

**Authors:** David E. Cohn, Aisling Forder, Erin A. Marshall, Emily A. Vucic, Greg L. Stewart, Kouther Noureddine, William W. Lockwood, Calum E. MacAulay, Martial Guillaud, Wan L. Lam

**Affiliations:** ^1^ Department of Integrative Oncology, British Columbia Cancer Research Centre, Vancouver, BC, Canada; ^2^ Department of Biochemistry and Molecular Pharmacology, New York University (NYU) Langone Medical Center, New York, NY, United States

**Keywords:** tumor immune microenvironment (TIME), tumor microenvironment (TME), tumor-infiltrating lymphocytes, immune checkpoint blockade, non-cell autonomous interactions, multiplexed imaging

## Abstract

The growth and metastasis of solid tumours is known to be facilitated by the tumour microenvironment (TME), which is composed of a highly diverse collection of cell types that interact and communicate with one another extensively. Many of these interactions involve the immune cell population within the TME, referred to as the tumour immune microenvironment (TIME). These non-cell autonomous interactions exert substantial influence over cell behaviour and contribute to the reprogramming of immune and stromal cells into numerous pro-tumourigenic phenotypes. The study of some of these interactions, such as the PD-1/PD-L1 axis that induces CD8^+^ T cell exhaustion, has led to the development of breakthrough therapeutic advances. Yet many common analyses of the TME either do not retain the spatial data necessary to assess cell-cell interactions, or interrogate few (<10) markers, limiting the capacity for cell phenotyping. Recently developed digital pathology technologies, together with sophisticated bioimage analysis programs, now enable the high-resolution, highly-multiplexed analysis of diverse immune and stromal cell markers within the TME of clinical specimens. In this article, we review the tumour-promoting non-cell autonomous interactions in the TME and their impact on tumour behaviour. We additionally survey commonly used image analysis programs and highly-multiplexed spatial imaging technologies, and we discuss their relative advantages and limitations. The spatial organization of the TME varies enormously between patients, and so leveraging these technologies in future studies to further characterize how non-cell autonomous interactions impact tumour behaviour may inform the personalization of cancer treatment.​

## 1 Introduction

The solid tumour microenvironment (TME) is a tumour-supporting niche that encompasses a diverse population of cells, including malignant, stromal, endothelial, bacterial, and immune cells. Within the larger TME, the population of immune cells is referred to as the tumour immune microenvironment (TIME). The TME is highly heterogeneous and can impact the progression of tumours, as well as their response to various therapeutics. The various cells of the TME influence tumour growth and invasiveness in numerous ways, including secreting cytokine factors that can activate cytotoxic effector cells, polarize immune cells, and drive both local and distal inflammation (1). The precise impacts of these cytokines on tumour biology vary between different cancer types and stages of tumour progression (1). Tumour microenvironments often have distinct physical and chemical traits, such as chronic hypoxia, which develops as a result of cancer cell growth outpacing the circulatory system’s capacity for expansion (2), and acidity, which arises largely as a consequence of high rates of glycolysis and the export of lactate and H^+^ ions by cancer cells (3). Many solid tumour microenvironments also contain distinct bacterial microbiomes, with taxonomic compositions that differ from those seen in healthy organs (4, 5).

Exposure to these unique characteristics of the TME, as well as to the cytokines and other signaling molecules secreted by malignant cells, frequently reprograms tumour-infiltrating and surrounding cells toward phenotypes and polarizations that further advantage the cancer cells. Reprogrammed stromal cells, such as cancer-associated fibroblasts (CAFs), create a highly cross-linked, stiff extracellular matrix (ECM) that promotes cell migration and invasion (6, 7). Reprogrammed cells of the TME, such as tumour-associated macrophages (TAMs), engage immunosuppressive programs that limit the anti-tumour activities of T cells (8). In this way there is extensive crosstalk through metabolites, cell surface receptor interactions, and secreted signaling molecules between cancer cells and other tumour-associated cells.

Specific features of an individual patient’s TME are now recognized to significantly impact their prognosis. Survival outcomes have been linked to a variety of factors, including diversity of species within the tumour microbiome (4), abundance or subtypes of CAFs (9, 10), or extent and distribution of tumour hypoxia (11), but the most well-described associations with patient outcomes are those pertaining to abundance, phenotypes, and distribution of immune cells both in the TME and within tumours themselves. DNA-, RNA-, and protein-level alterations acquired during malignant transformation can cause cancer cells to produce antigens with unique peptide sequences (“neoantigens”) (12, 13). Tumours that have greater genomic instability and a larger number of mutations (i.e. a higher mutational burden) are expected to display more neoantigens. Since neoantigens are not protected by central tolerance, their display on the surface of cancer cells can lead to T cell recognition and precipitate an anti-tumour immune response that improves patient outcomes (12, 14). Consequently, high quantities of tumour-infiltrating CD8^+^ T cells, and of cells that stimulate their function (e.g. CD4^+^ T cells, conventional type 1 dendritic cells, and B cells), are generally associated with relatively good prognoses (15–18). Conversely, large quantities of regulatory T cells (Tregs), myeloid-derived suppressor cells (MDSCs), and TAMs, all of which broadly impede anti-tumour immune responses, have been linked to poor prognoses in multiple cancer types (19–21). Furthermore, low levels of CD8^+^ T cells increase the risk of recurrence or progression for ductal carcinomas *in situ* and oral leukoplakias, suggesting that the composition of the immune cell infiltrate may also be predictive of the behaviour of precancerous lesions (22, 23).

Therapeutic strategies that target the TME, including immune checkpoint blockade (ICB) therapies, have profoundly impacted cancer patient survival rates, due in large part to their remarkable efficacy and even curative capability in some of the deadliest and most prevalent cancer types, such as melanoma and lung cancer. However, ICB induces responses in only a minority of patients, and is broadly ineffective against tumours that evade immune surveillance by preventing the generation or infiltration of tumour-specific T cells (24, 25). Patient responses to more widely-used treatments such as chemotherapy, radiation therapy, and various targeted therapies can also be impaired by TME-related factors, including low quantities of immune cell infiltration, dense stroma and poor circulation, and large areas of chronic hypoxia (2, 26, 27). The tremendous variance in the spatial organization of the TME between patients, even those with tumours driven by the same oncogene(s), remains a major confounding factor to treatment decisions and clinical outcomes. As an example, the confinement of immune cells to the tumour stroma and their exclusion from the tumour core has been associated with a decreased likelihood of response to anti-PD-L1 therapy (28), as well as poorer overall survival (29).

The spatial organization of TME-resident cells also influences the frequencies of non-cell autonomous interactions. These interactions can be driven by cell-cell contact, secreted proteins, or metabolites, and they contribute to cancer phenotypes. However, many methods used to study the TME, such as flow cytometry, only provide information about the abundance of cell subtypes and do not capture their spatial distribution. Imaging-based methods such as multiplex immunofluorescence (mIF) do record these spatial data, but the capacity for these technologies to deeply characterize interactions between specific cell subtypes is limited by their inability to visualize more than 6-8 markers in a given sample (30). Consequently, the recent creation of imaging technologies that enable highly multiplexed, high-resolution spatial profiling of solid TMEs is impactful, and has the potential to elevate the study of the malignant immune microenvironment from measuring cell abundance to investigating “cell sociology” – the myriad relationships, interactions, and communications between cells (31). In this article, we review the study of cell sociology in the TME through (a) summarizing the well-described non-cell autonomous interactions within the TME and their impact on tumour behaviour and therapeutic response, (b) discussing the image analysis programs and computational methods currently used to decipher cell sociology in the TME, and (c) reviewing recently developed highly-multiplexed spatial imaging technologies.

## 2 Non-cell autonomous interactions within tumors

### 2.1 Non-cell autonomous interactions mediated by cell-cell contact

The development of multiplex immunohistochemistry (mIHC) and mIF has made it possible to quantify how often cells of different types exist close to each other in the tumour microenvironment and consequently to estimate the frequency of specific cell-cell contacts. As we will discuss, physical contacts between cancer, immune, and bacterial cells within the TME play significant roles in the modulation of the anti-tumour immune response and cancer cell proliferation (**Supplementary Table 1**).

CD8^+^ T cells are among the most critical immune components of the TME, due to their ability to recognize, bind, and kill neoantigen-expressing tumour cells. However, tumour cells are only susceptible to this destruction if they come into direct contact with CD8^+^ cells. In metastatic colon cancer, having a high percentage of tumour epithelial cells located in proximity to CD8^+^ T cells has been linked to increased survival, despite overall CD8^+^ T cell infiltration being lower in these patients’ tumours (32). Similarly, the likelihood of lung adenocarcinoma recurrence has been observed to correlate more strongly with the frequency with which CD8^+^ cells neighbour tumour epithelial cells than with the overall CD8^+^ cell density (31). Underscoring the importance of immune cell subtyping, relapse-free survival in triple-negative breast cancer (TNBC) has been linked specifically to high levels of CD8^+^CD103^+^ T cells in immediate proximity to cancer cells (33). CD8^+^ T cell function is also mediated by other immune cells; for example, an increased proximity of CD8^+^ T cells to CD3^+^CD8^-^FOXP3^-^ helper T cells (32) and B cells (34) has been associated with improved patient outcomes.

Direct interactions between T cells, B cells, and dendritic cells occur in tertiary lymphoid structures (TLSs), which are dense clusters of immune cells commonly present in chronically inflamed areas, including the tumour microenvironments of multiple cancers (35). TLSs contain both T and B cell regions and are transient sites of the germinal centre reactions, which lead to B cell differentiation (35). T follicular helper (Tfh) cells are significant components of TLSs, being involved in both TLS formation and the germinal centre reactions (36, 37). While the production of IL-21 and CXCL13 by Tfh cells can stimulate adaptive anti-tumour immunity at a distance (38), the direct engagement of Tfh cells with B cells through ICOS/ICOSL and CD40L/CD40 binding is also critical to the anti-tumour immune response (39).

**Figure 1:**
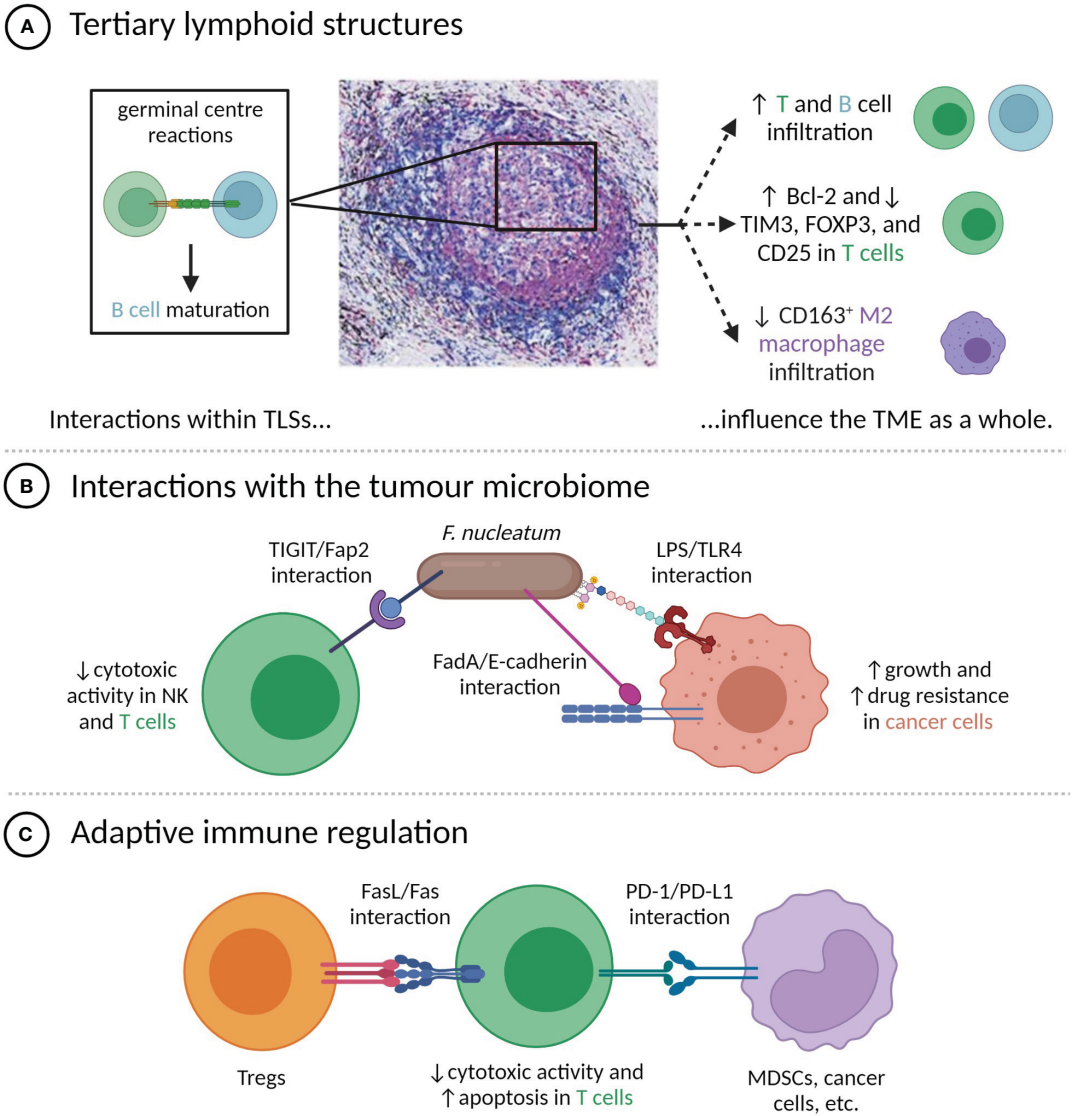
Direct contacts between cells in the TME modulate tumour phenotypes. **(A)** Cell-cell interactions within TLSs, particularly the germinal centre, influence TME composition and immune phenotypes. TLS image is reproduced from (52) with permission of the authors; **(B)** Interactions between *F nucleatum* and TME-resident cells have pro-tumour effects; **(C)** T cell anti-tumour activity is attenuated by direct contact with Tregs and PD-L1^+^ cells.

The presence of TLSs has been linked to improved prognoses in a number of cancers, including melanoma (40), head and neck squamous cell carcinoma (41), pancreatic ductal adenocarcinoma (PDAC) (42), and muscle-invasive bladder cancer (43), and can have greater prognostic value than the bulk count of infiltrating CD8^+^ T cells (44–46). Tumours with TLSs tend to have distinct features, including increased infiltration by CD20^+^ B cells and both CD8^+^ and CD4^+^ T cells (44, 47), decreased infiltration of CD163^+^ M2 macrophages (48), decreased expression of the Treg markers FOXP3 and CD25 by CD4^+^ T cells (48, 49), and higher levels of Bcl-2 and lower levels of TIM3 expressed by T cells (40). These associations may in part reflect an increased likelihood of TLS development in the presence of chronic inflammation caused by a pre-existing anti-tumour immune response, as suggested by Cabrita et al. (40), but are also due to the local amplification of that response by TLSs through recruiting lymphocytes and facilitating antigen presentation and lymphocyte maturation (35, 50, 51) (**Figure 1A**). Supporting the idea that TLSs have a regional impact on the immune response, shorter distances between TLSs and the invasive front of bladder tumours have been linked to increased disease-specific survival (43). There is also some evidence that intra-tumoural TLSs are more prognostically favourable than peri-tumoural TLSs (48, 53).

Tumour-resident bacteria within the TME can influence tumour phenotypes in numerous ways, including through cell-cell contacts with cancer and immune cells. One of the best-characterized examples is *Fusobacterium nucleatum*, a Gram-negative bacterium that is normally present in the oral cavity, but that has been found within the TME of a number of cancer types, most prominently colorectal cancer (CRC) (54) (**Figure 1B**). The Fap2 protein of *F. nucleatum* can bind the immune checkpoint protein TIGIT, commonly expressed by NK and T cells, limiting their cytotoxic activity (55). *F. nucleatum* also expresses FadA adhesin, which can bind the E-cadherin expressed by CRC cells. This induces the nuclear translocation of β-catenin and downstream expression of oncogenic proteins, including Wnt, Myc, and cyclin D1, which stimulate tumour growth (56). *F. nucleatum* additionally exerts a number of pro-tumour effects through interactions between lipopolysaccharide (LPS) and host-expressed TLR4, including upregulation of hsa-miR-21-5p, which stimulates cancer cell growth and invasion (57), and downregulation of hsa-miR-18a-3p and hsa-miR-4802, which induces autophagy and confers resistance to oxaliplatin and 5-fluorouracil (58).

### 2.2 Influence of cell-cell contacts on immunotherapy response

As mentioned, the targeting of the PD-1/PD-L1 axis by ICB therapy represents a breakthrough in cancer therapy for thousands of patients, but the majority of treated patients do not experience responses (**Figure 1C**) (59). It has been frequently proposed that response to ICB is more common in the case where PD-L1 expression is not simply constitutive, but is instead induced by cytokines produced during an adaptive immune response (25, 60). Proxies for the presence or likelihood of an adaptive immune response, including tumour mutational burden, PD-L1 expression, various signatures of inflammatory gene expression, and biomarkers based on mIHC or mIF have all been correlated with response to anti-PD-1 or PD-L1 therapy, with a recent meta-analysis by Lu et al. finding mIHC/mIF biomarkers to be the most accurate predictors of therapeutic response (61). These spatial biomarkers offer unique insight into whether PD-L1^+^ cells are enriched in the vicinity of functional targets (in which case inhibition of the PD-1/PD-L1 axis is thought to be more likely to amplify anti-tumour immunity) (60) or randomly distributed throughout the TME, and can only be measured through multiplexed imaging. For instance, high levels of proximity between PD-L1^+^ cells and either PD-1^+^ cells (25, 62, 63), exhausted CD8^+^ cells (64), or cancer cells (65) have been associated with improved outcomes after ICB therapy. Similarly, high expression of PD-L1 by M1 macrophages that were located near both CD8+ T cells and the tumour-stroma boundary has been correlated with increased likelihood of response of metastatic melanoma to ICB therapy (66).

While adaptive expression of PD-L1 may represent a therapeutic opportunity, the PD-1/PD-L1 interaction is not uniformly associated with improved patient outcomes. Frequent interactions between PD-L1^+^ cells and CD8^+^ cells or PD-1^+^ cells have been correlated with poor outcomes in HPV^-^ oral and oropharyngeal squamous cell carcinomas, while overall PD-L1^+^ abundance has not (67, 68). Interestingly, a study of diffuse large B cell lymphoma found that PD-1^+^/PD-L1^+^ interactions were associated with poor outcomes in patients with high infiltration of CD3^+^ cells, but improved outcomes in patients with low CD3^+^ infiltration (69). These improved outcomes may arise because PD-1^+^/PD-L1^+^ interactions indicate that the few CD3^+^ cells present are concentrated near tumour cells, which, despite the consequent immune checkpoint activation, is more favourable than them being isolated (69). This highlights how the prognostic interpretation of cell-cell interaction scores should be informed by the overall immune context of the tumour.

### 2.3 Non-cell autonomous interactions mediated by intermediaries

CD4^+^ and CD8^+^ regulatory T cells generally suppress immune responses, and consequently, their infiltration into tumours has been linked to worsened prognoses in many forms of cancer (19). Tregs attenuate anti-tumour immune activity through a variety of mechanisms, including depleting IL-2 from the TME (70), pushing antigen-presenting cells (APCs) towards tolerogenic phenotypes characterized by the downregulation of CD80/86 and the upregulation of IDO1 (71), inducing apoptosis in CD8^+^ cells via expression of the Fas ligand (72) or granzyme and perforin (73), and secreting immunosuppressive cytokines, including IL-10 and TGF-β (74). Links to poor outcomes have been observed for Tregs located near CD8^+^ T cells in HPV^-^ oral squamous cell carcinoma (OSCC) (68) and in CRCs with microsatellite instability (75), and for both CD4^+^ and CD8^+^ Tregs located near non-small cell lung cancer (NSCLC) cells (76, 77). However, increased proximity between Tregs and CD8^+^ T cells has been associated with improved prognoses in gastric tumours and NSCLC (76, 78). Taken together, these results suggest not only that Treg/CD8^+^ interactions have distinct functional repercussions in different cancer types, but also that the dominant functions of tumour-infiltrating Tregs depend on their immediate immune context. As a further example of this, mouse models suggest that lung TLSs commonly contain Tregs, which limit the activation and proliferation of the TLS-localized T cells (79). A study of TLS-localized Tregs in breast cancer linked their presence to poorer patient outcomes, despite finding that the presence of Tregs in the tumour bed had no significant prognostic value (80).

High densities of TAMs are associated with poor outcomes in many types of cancer (21), owing to a range of functions that includes the secretion of growth factors, survival factors, and immunosuppressive cytokines (81). The impact of these growth and survival factors has been observed in NSCLC, where cancer cells undergoing apoptosis were on average located closer to pro-inflammatory M1-polarized macrophages than to anti-inflammatory M2-polarized macrophages, while the reverse was true for proliferative Ki67^+^ cancer cells (82). Accordingly, higher numbers of cancer cells located in proximity to M2 macrophages were found to be associated with poor patient outcomes, while the opposite was true for M1 macrophages (82). High M2 macrophage/cancer cell proximity has been correlated with poor outcomes in PDAC (83), although in gastric cancer it has been linked to improved outcomes (84). M2 macrophages can also engage CD8^+^ T cells in lengthy, antigen-specific interactions that do not instigate T cell proliferation, but instead induce an exhausted, PD-1^+^ phenotype (85). Short distances between CD8^+^ T cells and HLA-DR^-^ (predominantly M2) macrophages have been associated with decreased survival in melanoma, potentially due to the immunosuppressive nature of the CD8^+^/M2 interaction (86).

### 2.4 Generation of pro-tumour metabolite profiles in the TME

Cells within the TME can induce pro-tumour metabolite profiles in a number of ways, including depleting metabolites required for the cytotoxic activity of immune cells, secreting immunosuppressive metabolites, and secreting metabolites that stimulate cancer cell growth and division.

**Figure 2:**
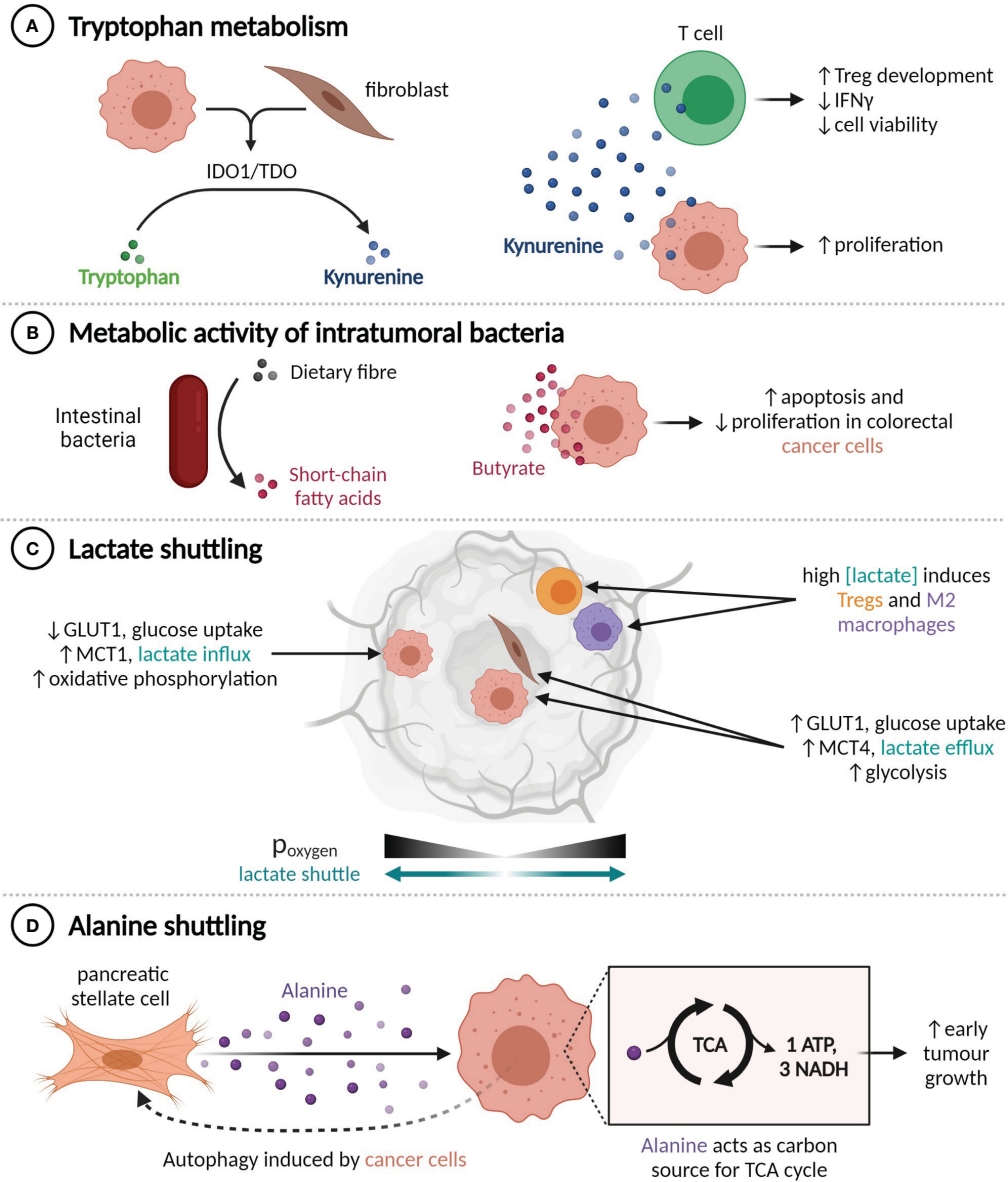
TME metabolite profiles alter tumour behaviour. **(A)** Production of kynurenine by IDO1^+^ or TDO^+^ cells enhances cancer cell proliferation and impairs T cell functionality. **(B)** Conversion of dietary fibre into butyrate by intestinal bacteria suppresses colorectal cancer cell proliferation. **(C)** Lactate shuttling is induced by oxygen gradients, promoting resistance to angiogenesis-inhibiting treatments. **(D)** Pancreatic cancer cells induce autophagy in nearby pancreatic stellate cells, liberating alanine, which the cancer cells use to fuel the TCA cycle.

Within the TME, the catabolic action of the IDO1 and TDO enzymes can convert the amino acid tryptophan into kynurenine (87, 88). Kynurenine engenders an immunosuppressive environment by promoting Treg development and decreasing the viability and IFNγ expression of CD8^+^ T cells, while also directly increasing cancer cell proliferation (**Figure 2A**) (89–91). IDO1 and TDO can be expressed constitutively by cancer cells, in which case they inhibit lymphocyte infiltration, or expressed by cancer and stromal cells in response to IFNγ, which limits the activity of tumour-infiltrating lymphocytes (92). A number of IDO1 inhibitors have been developed, but initial phase III trials have not shown any clinical benefit from adding IDO1 inhibitors to anti-PD-1 therapy regimens (93). It has been suggested that the relative benefit of IDO1 inhibition may be greater in immune-cold tumours that constitutively express IDO1 (94), as it could enable increased lymphocyte infiltration and in turn amplify the utility of PD-1 or PD-L1 inhibition.

The metabolic activity of commensal bacteria can also influence the tumour microenvironment. Intratumoural bacteria that express the long isoform of cytidine deaminase, such as *Enterobacteriaceae*, are found commonly in human PDACs and have been shown to convert the chemotherapeutic agent gemcitabine into an inactive form, which confers drug resistance in mouse models (95). Conversely, various species of gut bacteria participate in the conversion of primary bile acids to secondary bile acids and the fermentation of dietary fibre into short-chain fatty acids (SCFAs). The SCFA butyrate, which is exclusively synthesized by the gut microbiome, has histone deacetylase inhibitor activity at high concentrations and is known to inhibit CRC cell proliferation and promote apoptosis through a range of mechanisms, including upregulating miR-203 (96), limiting ERK phosphorylation (97, 98), and upregulating p21 (**Figure 2B**) (97).

The metabolic composition of the TME is also altered by hypoxia, as it prevents cells from obtaining energy through oxidative phosphorylation and induces, among other transcriptional programs and adaptive phenotypes (99), compensatory upregulation of the glucose importer GLUT1 and the lactate exporter MCT4 (100). Expression of GLUT1 and MCT4 is thus highest in tumour regions that are far from blood vessels (101). This metabolic shift results in an excess of lactate within the TME, which can then be imported by MCT1-expressing cells within normoxic tumour regions and used as an energy source or anabolic building block (102–104) (**Figure 2C**). This lactate shuttle facilitates the survival of cancer cells in hypoxic regions through decreasing the glucose requirements of cells in nearby normoxic neighbourhoods (102). Establishment of this shuttle is an observed mechanism of resistance to angiogenesis-inhibiting therapy in a number of tumour types (105–107). The secretion of lactate by cancer cells also contributes to the reprogramming of immune cells, including through promoting the M2 polarization in macrophages (108, 109) and augmenting Treg induction (110).

Interactions between cancer cells and CAFs can induce HIF-1 and redox-mediated expression of MCT1 in cancer cells and both GLUT1 and MCT4 in CAFs, thereby creating a CAF-cancer cell lactate shuttle (103, 111, 112). Cancer cells involved in this shuttle generally exhibit MCT1-dependent increases in proliferative (103, 113, 114), invasive (115, 116), and migratory (116) capacity. Ippolito et al. have shown that the exposure of prostate cancer cells to CAF-conditioned media increases their mitochondrial mass and oxygen consumption, which were further augmented by the uptake of mitochondria from neighbouring CAFs along cytoplasmic bridges (115). Prostate cancer patients with both MCT1^high^ cancer cells and MCT4^high^ stromal cells tend to have later stage (pT3) tumours (117), and experience poor, stage-independent, biochemical failure-free survival (118). Neither of these associations were seen in patients with high expression of only one MCT (117, 118), which highlights the clinical significance of lactate shuttling.

Similarly, an alanine shuttle is induced by interactions between pancreatic stellate cells (PSCs) and PDAC cells. Exposure to PDAC-conditioned media causes PSCs to undergo autophagy and secrete alanine, which can be imported by PDAC cells and used as a carbon source for the synthesis of TCA cycle metabolites (119) (**Figure 2D**). Knockdown of the alanine importer *SLC38A2* abrogates the increase in tumour growth seen when PSCs are injected into mouse xenograft models in addition to PDAC cells, which suggests that alanine shuttling is responsible for this growth advantage (120).

## 3 Emerging approaches for quantifying cell-cell interactions

**Figure 3:**
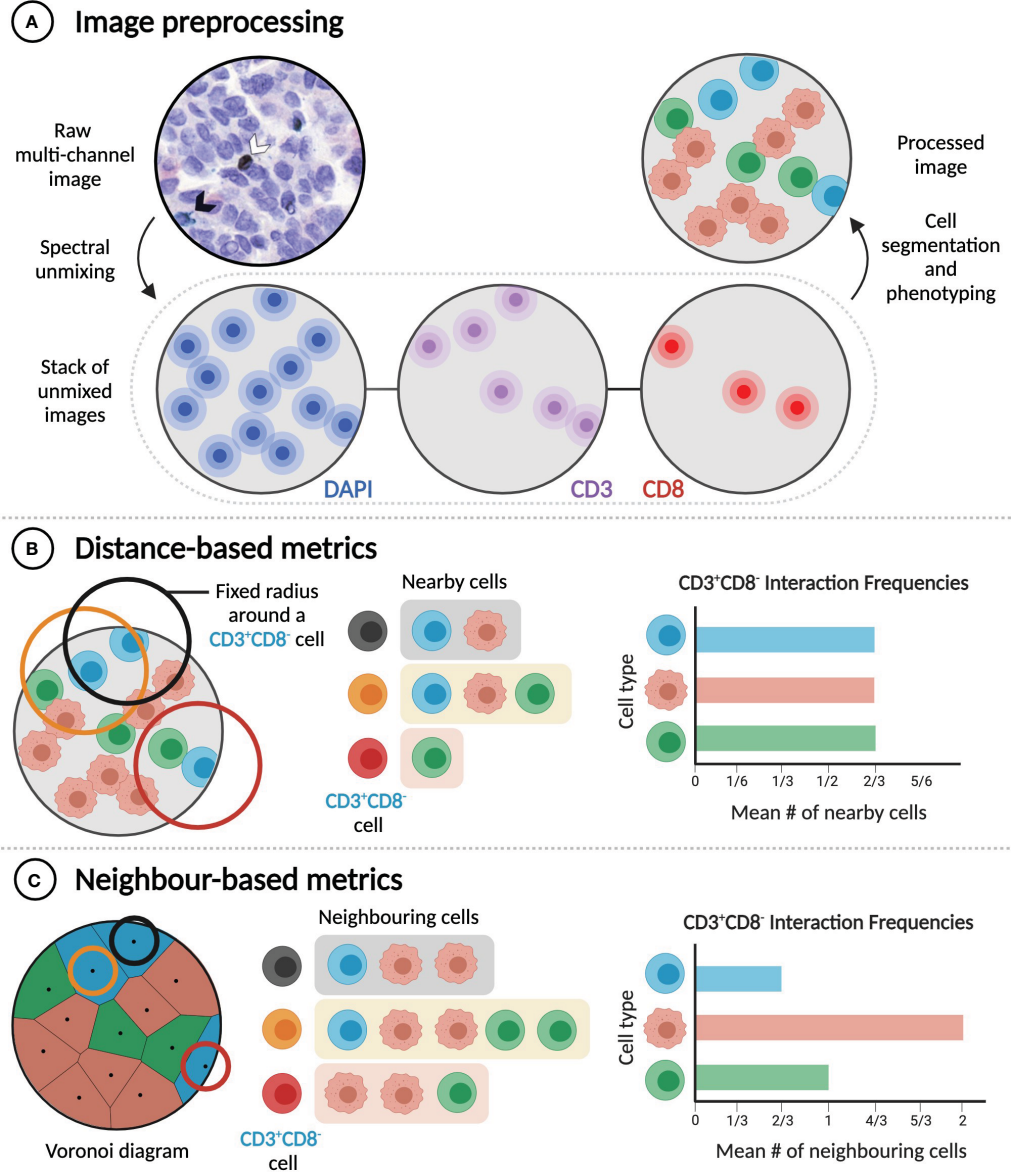
Distance and neighbour-based methods quantify cell-cell interactions differently. **(A)** Extracting rates of cell-cell interactions from imaging data requires image preprocessing, which includes cell segmentation and phenotyping. Raw multi-channel image reproduced from (31) with permission of the authors. **(B)** Distance-based metrics calculate interaction frequencies by considering a pair of cells to interact if they are in physical proximity (e.g. within a given radius of one another). **(C)** In contrast, neighbour-based metrics consider a pair of cells to interact if they are neighbours (e.g. if they share a Voronoi edge), regardless of the distance between them. In the examples illustrated here, frequencies of interaction between CD3^+^CD8^-^ cells and three different cell types are calculated.

Most of the multiplexed spatial data collected from tumour samples is in the form of multi-channel images derived from mIHC or mIF, imaged by either absorption or fluorescence microscopy. These images must undergo processing, typically including spectral unmixing, selection of regions of interest (ROIs), nuclear and cell segmentation, and cell phenotyping (**Figure 3A**) (30).

Cell segmentation and phenotyping performed during image processing enables subsequent extraction and analysis of cell sociology features, most notably the extent of interaction between different cell types (e.g. A^+^ and B^+^ cells). Cell-cell interactions are most commonly quantified using metrics that depend on intercellular distances, including the mean or median distance from an A^+^ cell to the nearest B^+^ cell (32, 65, 77, 86), the absolute number of B^+^ cells located within a specified distance of one or more A^+^ cells (63, 67, 68, 82, 121), the percentage of A^+^ cells with a B^+^ cell located within a specified distance (32, 84), and the area under the curve of the A^+^-B^+^ G-cross function (76, 122) (**Figure 3B**). The choice of metric, which can also require choosing a specific threshold distance (commonly between 10 and 40 µm) (32, 63, 67, 68, 82, 84, 121), and the decision of whether to apply cell density normalizations should be guided by the specific biological hypothesis being tested. Distance-based metrics are generally useful for determining the frequency of particular short-range cell-cell interactions in a sample, but they are influenced by cell size and do not identify direct contacts between cells.

The second class of metrics used to quantify the frequency of interaction between cell types can be referred to as ‘neighbour-based’ (**Figure 3C**). Starting from the positions of cell nuclei, Voronoi diagrams, or equivalently Delaunay triangulations, can be constructed to identify pairs of cells that are immediate neighbours and are likely in physical contact (31, 123). Alternatively, a cell’s neighbours can be identified through dilating its binary mask (62, 69). Neighbour-based metrics include the mean fraction of the neighbours of A^+^ cells that are B^+^ (31); the number of B^+^ cells that neighbour at least one A^+^ cell, normalized to the total number of immune cells (62, 69); and the ratio of the number of observed A^+^-B^+^ neighbour pairs to the expected number of such pairs, based on the proportions of all imaged cells that are A^+^ or B^+^ (123). These metrics are less dependent on cell size, but they are not suitable for the analysis of cell-cell interactions that do not require direct contact, and careful specification of ROIs is necessary to avert analysis artifacts, such as cells in isolated areas of a slide being assigned unreasonably large volumes (31). While they aim to measure the same quantity, distance-based and neighbour-based metrics can yield significantly different results (**Figures 3B, C**). Both classes of metrics have advantages in particular cases, and so the best metric for a given experiment depends on the hypothesis being tested.

With the growth of digital pathology, many bioimage analysis programs, both open and closed-source, have been developed to analyze these metrics (**Table 1**). In the open-source domain, in addition to ImageJ, researchers now have access to a range of alternatives, including QuPath, CellProfiler, Icy, MCMICRO, Cytomapper, and others (**Table 1**) (124–129). Open-source tools have become increasingly important, as they allow users to develop, customize, and share their own analysis solutions, commonly in the form of plug-ins, which makes these programs highly flexible and stimulate innovation (130). Closed-source software generally does not permit users to modify, augment, or share the software, but it frequently possesses superior ease of use, available online support, and vertically integrated support for common analysis tasks. HALO (Indica Labs, Albuquerque, NM, USA) and Visiopharm (Visiopharm A/S, Hørsholm, Denmark) are examples of closed-source tools, but there are a variety of others, frequently integrated with image acquisition hardware such as Aperio image analysis (Leica Biosystems, Buffalo Grove, IL, USA) and TissueGnostics Quest suites (TissueGnostics, Vienna, Austria) (**Table 1**). Each image analysis program offers its own advantages, and identifying the most suitable program for a given analysis project requires consideration of a number of factors, including project objectives, the scale of required analysis, and user experience and training. More recently, programs that focus on the statistical analysis of spatial cell distributions within tissues (cell neighbourhood analysis) have been developed. Many of these (SPIAT, Giotto, histoCAT/NeighbouRhood) (131–134) take only the spatial location of the cells and their quantified characteristics (such as protein marker expression and RNA sequence counts) as input and are predominantly written in R, allowing for user-specific optimization and modifications. Generally, these programs allow the user to define the size (in the number of cell layers, the distance from the cell, or both) of the neighborhood that they want to consider. Giotto in particular allows users to interactively refine cell marker positivity based upon neighbourhood characteristics. As these newer programs demonstrate, the capability of programs available for digital pathology analysis continues to expand, giving researchers access to a constantly evolving toolkit to meet the computational demands of highly-multiplexed imaging technologies.

**Table 1:** Characteristics of selected bioimage analysis programs.

Software Name	Open Source	Base Language	Utility and Notable Features*	AI Modules	WSI
ImageJ/FIJI	Open	Java	Comprehensive low level image processing; many user-developed macros and plug-ins	No	No
QuPath	Open	JavaFX	Tissue image analysis: stain and cell quantification, etc.; batch processing using user-created extensions and workflows	Yes	Yes
CellProfiler 4.0	Open	Python	High-throughput cell image analysis; customizable, modular pipelines for image analysis; user-developed modules	No	No
Cytomapper	Open	R	Visualization of pixel- and cell-level information; input single-cell expression values and cell-specific metadata from highly multiplexed imaging; Bioconductor package	Yes	No
Icy	Open	Java	Comprehensive image analysis platform; graphical programming interface for workflow design; many user-developed plug-ins; dependency management	No	No
HALO	Closed	–	Tissue image analysis; automated tissue classification; modular workflow; bespoke modules for cell-cell analyses	Yes	Yes
Visiopharm	Closed	–	AI-based tissue image analysis; automated cell phenotyping; high-throughput TMA analysis	Yes	Yes
Aperio	Closed	–	Tissue image analysis; variety of algorithmic tools for quantification of multiplex images	Yes	Yes
TissueGnostics Quest suites	Closed	–	Tissue image analysis; automatable macros and pre-made apps; compatible with ImageJ and MATLAB scripts; import tools for wide range of image formats	Yes	Yes
MCMICRO	Open	Any (Nextflow and Galaxy)	Tissue image analysis and spatial neighborhood image analysis; input multiplexed WSI, TMA; optional segmentation step; run multiple algorithms in parallel; use of software containers makes it interoperable with any programming language	Yes	Yes
SPIAT	Open	R	Spatial neighborhood image analysis; input is cell location/coordinates and characteristics; automated detection of cellular neighbourhoods; fast processing	Yes	No
Giotto	Open	R	Spatial neighborhood image analysis; input is cell location/coordinates and characteristics; interactive Giotto viewer	Yes	No
histoCAT/NeighbouRhood	Open	R	Spatial image analysis of cells in tissues; uses ‘CellProfiler output’ or ‘CellProfiler output’-like data; visualization of images and single-cell analysis in parallel; graphical user interface (histoCAT) and R implementation (neighbouRhood)	Yes	No

*All listed programs are compatible with both brightfield and fluorescence images.

WSI, whole slide imaging; TMA, tissue microarray.

## 4 Advantages and applications of highly-multiplexed imaging technologies

**Figure 4:**
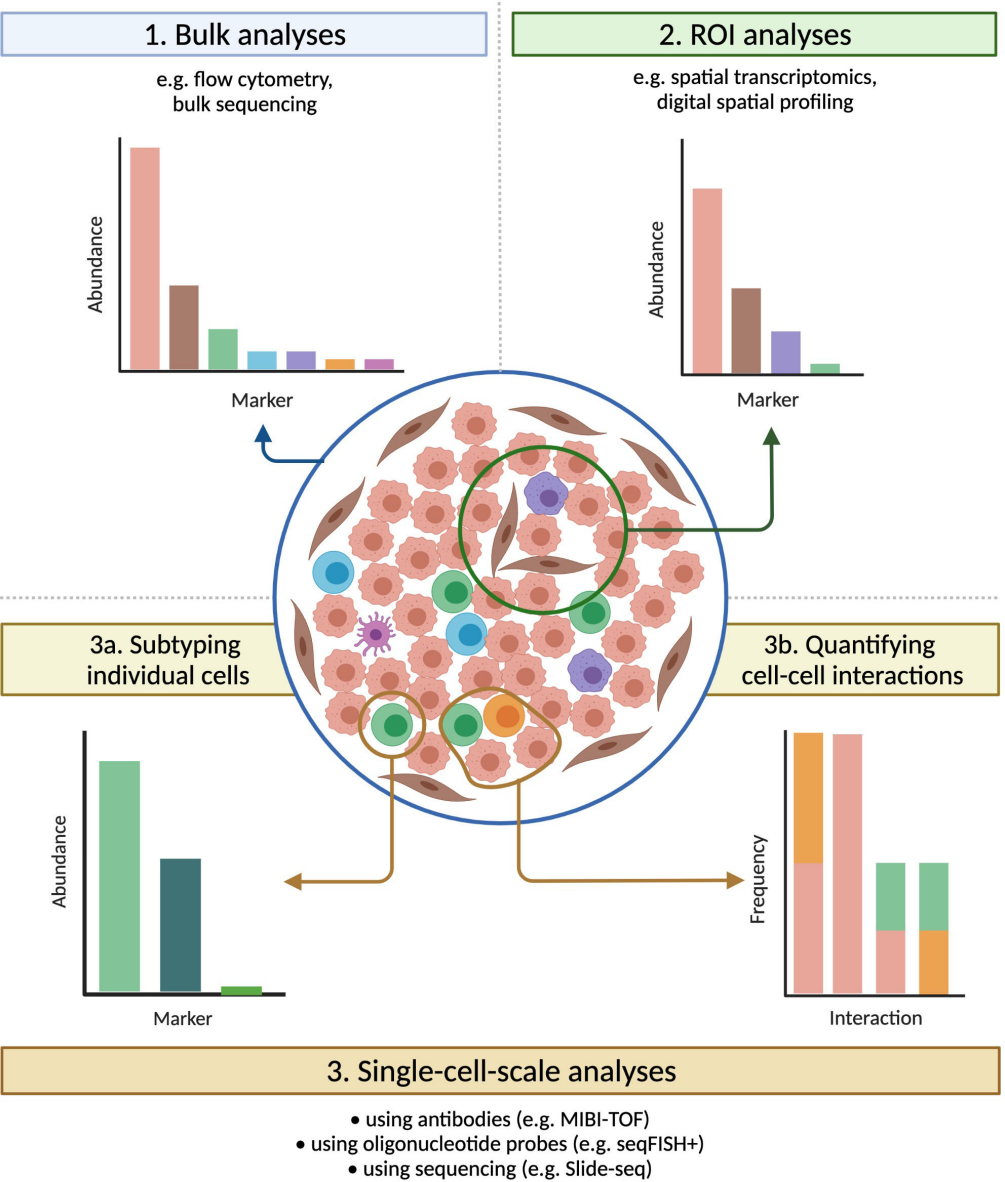
Technologies that preserve spatial information have broad applications. Tumours that carry identical densities of immune cells, and that would appear similar if evaluated by bulk methods, can vary significantly in their spatial organization. Imaging technologies provide a bevy of information on samples, both within multiple-cell ROIs (useful for e.g. assessing intra-tumour heterogeneity) and at the single-cell scale (ideal for e.g. subtyping individual cells or quantifying cell-cell interactions), greatly expanding the range of possible analyses.

As discussed above, the advent of mIHC and mIF has greatly enhanced our understanding of non-cell autonomous mechanisms within the tumour microenvironment. Imaging with a fluorescence microscope can achieve sub-micrometer resolutions, and the use of secondary antibodies and tyramide signal amplification allows for the detection of even lowly-expressed molecular species (135). Yet even with the application of increasingly sophisticated computational analysis, the width and consequent overlapping of the spectra of common fluorophores makes the simultaneous fluorescence imaging of more than 6-8 markers infeasible (30, 136). The TME commonly contains a wide variety of cell types, which may be reprogrammed into various phenotypes or polarizations, and so many relevant questions cannot be answered with only these 6-8 markers. Recently developed imaging technologies can achieve considerably higher levels of multiplexing, which greatly expands their potential applications, but each technology has its own advantages and disadvantages in areas such as imaging resolution, sensitivity, sample throughput, and sample integrity (**Figure 4**, **Tables 2**, **3**).

**Table 2:** Technologies for spatial analysis of protein expression in the TME.

Technology	Antibody Conjugation	Multiplexing* (proteins)	Spatial Resolution	Sensitivity	Reference
mIF/mIHC	Fluorophore or chromogen	8	DL; SR-compatible	2° antibodies usable	(135)
t-CyCIF	Fluorophore	≥ 60	DL; SR-compatible	Use of 2° antibodies limited	(137)
CODEX	Oligonucleotide	≥ 56	DL	Use of 2° antibodies limited	(123)
DEI	Oligonucleotide	≥ 8	DL; SR-compatible	Use of 2° antibodies limited	(138)
Exchange-SABER	Oligonucleotide	≥ 10	DL; SR-compatible	5 to 180-fold increase in 1° antibody signal	(139)
MIBI-TOF	Lanthanide	≥ 36	Comparable to DL	Near single-antibody	(140)
DSP	Oligonucleotide	≥ 44	Defined by choice of ROI	Use of 2° antibodies limited	(141)

*Value indicates the highest level of multiplexing that has been demonstrated.

DL, diffraction-limited resolution; SR, super-resolution.

**Table 3:** Technologies for spatial analysis of RNA expression in the TME.

Technology	RNA-Binding Probes	Multiplexing* (RNAs)	Spatial Resolution	Detection Threshold	Reference
DSP	Oligonucleotide-conjugated oligonucleotides	≥ 2093	Defined by choice of ROI	~600 copies of a transcript	(141)
seqFISH+	Barcoded oligonucleotides	≥ 10,000	SR	~2-fold higher than smFISH	(142)
STARmap	Primer and padlock probes	≥ 1020	DL; diminished by RCA	Comparable to scRNAseq	(143)
HiPR-FISH	Barcoded oligonucleotides	≥ 65 bacterial taxa	Single-cell	~790 ribosomes per cell	(144)
FISSEQ	RT primers containing random hexamers	Whole transcriptome	DL; diminished by RCA	~200-400 copies of a transcript	(145)
ST	Spatially barcoded oligo(dT) probes	Whole transcriptome	100 µm	~14-fold higher than smFISH	(146)
HD-ST	Spatially barcoded oligo(dT) probes	Whole transcriptome	2 µm	~77-fold higher than smFISH	(147)
Slide-seqV2	Spatially barcoded oligo(dT) probes	Whole transcriptome	10 µm	~2-fold higher than scRNAseq	(148)

*Value indicates the highest level of multiplexing that has been demonstrated.

DL, diffraction-limited resolution; SR, super-resolution.

### 4.1 Antibody-based methods

Cyclic immunofluorescence, which broadly involves repeated stain-image-bleach/wash cycles being performed on the same tissue sample, is the simplest extension of mIF. One of the furthest developed forms of this technology is t-CyCIF, in which each imaging cycle visualizes three fluorophore-conjugated primary antibodies and a DNA dye (137). Sample integrity declines with successive imaging cycles, but 60-fold multiplexing has been successfully demonstrated (137). t-CyCIF achieves this multiplexing at high, diffraction-limited resolutions and does not require a specialized microscope, but each imaging cycle is 6-8 hours long (137). Other variants of cyclic IF elute antibodies instead of photobleaching fluorophores (149). This enables the use of secondary antibodies, but harsh elution conditions can alter the structure of some epitopes, and so careful optimization of staining order and antibody choice is necessary (149).

Co-detection by indexing (CODEX) imaging is another cyclic technique, and involves staining with antibodies that are conjugated to oligonucleotide tags with 5’ overhangs of different lengths, followed by multiple cycles of imaging (123). In each cycle, a mix of unlabelled (‘indexing’) and fluorescently labelled dNTPs is added, causing each type of labelled dNTP to be incorporated into a unique tag. CODEX was later re-engineered so that each imaging cycle instead involved the addition of three fluorophore-conjugated oligonucleotides, each complementary to a region on a single antibody’s tag (150). This newer version of CODEX has demonstrated 56-fold multiplexing, and can be performed using a standard fluorescence microscope (150). However, species limitations make the use of secondary antibodies impossible at these high levels of multiplexing, and so imaging sensitivity is limited.

Similar to CODEX, DNA-Exchange-Imaging (DEI) is a cyclic technique in which a fluorophore-conjugated oligonucleotide is introduced during each imaging cycle, and binds to a single antibody-conjugated oligonucleotide (138). DEI is compatible with super-resolution platforms (138), but it shares CODEX’s drawback that the expansion of multiplexing beyond levels achievable through mIF makes the use of secondary antibodies impossible.

Exchange-SABER combines DEI with immunostaining with signal amplification by exchange reaction (Immuno-SABER). In Immuno-SABER, each antibody-conjugated oligonucleotide is bound by a DNA concatemer that has been synthesized via primer exchange reactions. This hybridization creates a lengthy overhang that contains numerous binding sites for short, fluorophore-conjugated ‘imager’ oligonucleotides (139). This approach allows for significant (5 to 180-fold) signal amplification, with the high-end being achieved when secondary and tertiary concatemers are used (Iterative-SABER) (139). Exchange-SABER holds the potential for high levels of multiplexing, but the imaging of more than 10 markers simultaneously has yet to be demonstrated (139).

Multiplexed ion beam imaging (MIBI) involves the preliminary staining of tissues with lanthanide-conjugated antibodies (151). During the imaging process a narrow primary ion beam is scanned across the sample pixel by pixel, which liberates secondary ions from the antibodies, allowing the lanthanide isotopes to be quantified by a mass spectrometer (151). MIBI-TOF, an improved version of MIBI that uses time-of-flight mass spectrometry, has demonstrated simultaneous imaging of up to 36 markers at sub-micrometer resolution, and can approach single-antibody sensitivity (140). However, despite improvements in MIBI-TOF’s throughput, achieving resolutions comparable to mIHC substantially increases the image acquisition time (140).

When used to assess protein expression, digital spatial profiling (DSP) employs antibodies that are conjugated via a UV-cleavable linker to unique oligonucleotides (141). After ROIs are determined, each is sequentially exposed to UV light, and the oligonucleotides released after each exposure are quantified through sequencing or the nCounter system (141). High levels of multiplexing (44 proteins) have been demonstrated, and the ability to establish ROIs of any shape allows for the profiling of highly specific areas of tissue (141). However, using very small (~single-cell) ROIs significantly impairs the detection of lowly-expressed proteins.

Collectively, these antibody-based techniques excel at providing information needed to answer questions about the prevalence, identity, and location of specific immune and stromal cell subtypes within the TME, and about how the proximity or interaction of different cell types impacts tumour behaviour. Demonstrated applications of these technologies include the identification of cellular phenotypes that are consistently located in close spatial proximity in the TNBC TME (121) and the screening of many protein markers simultaneously in distinct compartments of the non-small-cell lung cancer TME, in order to identify protein/compartment pairs that are associated with improved survival outcomes in patients treated with anti-PD-1 checkpoint blockade therapy (152). These technologies can also be combined with computational approaches that operate on a larger spatial scale through treating samples not only as mixtures of single cells but as aggregations of regions (cellular neighbourhoods) with distinct compositions and functions. For instance, the use of clustering and tensor decomposition techniques on CODEX data has enabled the characterization of a granulocyte-enriched neighbourhood within the CRC TME whose functional state, as defined by the expression of PD-1 and CD4 on T cells, was associated with patient outcomes (150). Another example is the use of a recently reviewed technique, imaging mass cytometry (153), to interrogate the TME of lung adenocarcinoma and identify spatial features from 5 µm sections that were predictive of recurrence (154). This group used the same technique to reveal cellular neighbourhoods that were associated with survival in glioblastoma, which were then used to identify a specific population of macrophages that were associated with long-term survival (155). Antibody-based techniques are a classical method to interrogate the spatial organization of the TME, and technological advances are facilitating highly-multiplexed applications to improve our understanding of this organization.

### 4.2 Oligonucleotide probe-based methods

While antibody-based protein analysis is suitable for many applications, antibodies against some proteins are challenging to create, and achieving whole-proteome multiplexing is currently unrealistic. Interrogating RNA expression instead, such as through the use of oligonucleotide probes, can circumvent these difficulties and streamline the investigation of transcript-level variations (141).

Conjugating the ‘indexing’ oligonucleotides used in DSP to single-stranded RNA probes instead of antibodies allows DSP to be used for the analysis of mRNAs (141). This method has achieved over 2000-fold mRNA multiplexing, but the detection of above-background signal for a given transcript requires around 600 copies of that transcript, and consequently most mRNAs can only be detected in relatively large (≥ 50 µm diameter) ROIs (140, 156).

seqFISH+ employs primary oligonucleotide probes that each contain a unique four-region barcode, with each region being complementary to a single ‘readout probe’ sequence (142). Imaging consists of four rounds, each with 20 hybridize-image-strip cycles in which three distinct readout probes, each conjugated to a different fluorescent dye, are hybridized to regions of the primary probes (142). Each primary probe’s barcode can be uniquely identified by the set of three readout probes that hybridize with it, one during each imaging round, with the final round used for correcting errors. In a given imaging cycle only 1 in 60 mRNAs is visualized, which minimizes optical crowding and enables the sub-diffraction limit localization of each mRNA molecule (142). seqFISH+ has demonstrated 10,000-fold multiplexing, with a sensitivity greater than that of single-cell RNA sequencing (scRNA-seq), but like most cyclic or sequential imaging methods, it requires lengthy workflow times (142).

In spatially-resolved transcript amplicon readout mapping (STARmap), DNA amplicons are constructed in hydrogel-embedded tissue through reverse transcription (RT), cDNA circularization, and rolling circle amplification (RCA), with primer and padlock probes used to prevent non-specific amplification (143). Each padlock probe is specific to a single gene and contains a unique five-base barcode, which can later be decoded by sequencing with error-reduction by dynamic annealing and ligation (SEDAL) (143). The degree of multiplexing depends on barcode length, and thus far 1020-fold multiplexing has been demonstrated at single-cell resolution, but high degrees of multiplexing limit STARmap’s sensitivity, due to the difficulty of optically resolving amplicons that are located in physical proximity (143). Under ideal conditions, STARmap’s sensitivity is comparable to that of scRNA-seq.

Oligonucleotide probes can also be used for the identification of bacterial taxa, through methods such as HiPR-FISH. In HiPR-FISH, several probes per interrogated taxon, each specific to the same unique sequence within that taxon’s rRNA, are first introduced to the sample (144). Each probe carries two flanking sequences, each complementary to one of ten fluorescent readout probes, chosen in such a way that each taxon’s probes collectively contain sequences complementary to a unique subset of the readout probes. After hybridization of the readout probes and fluorescence imaging, cells are segmented and then a machine learning classifier is used to assign each cell to a taxon based on its emission spectra (144). HiPR-FISH can potentially differentiate between 1023 distinct taxa in only one round of imaging, but notably it cannot detect unexpected or rare taxa for which probes were not designed (144).

### 4.3 Sequencing-based methods

The second major approach to spatially quantifying mRNA expression is to employ sequencing, either *in situ* or after spatially barcoding transcripts, which enables whole-transcriptome multiplexing.

With fluorescent *in situ* sequencing (FISSEQ), sequencing libraries are constructed *in situ* through a process of RT, cDNA circularization, and RCA (145). Cross-linking of aminoallyl dUTP residues introduced during RT prevents cDNA diffusion. Each amplicon is sequenced by oligonucleotide ligation and detection (SOLiD): sequencing primers are annealed, and fluorescently-tagged oligonucleotides are used to identify every fifth nucleotide (145). The primer is then stripped and the procedure is repeated with primers of incrementally shorter lengths, so that all nucleotides in the sequence are eventually ascertained. FISSEQ operates at single-cell resolutions, but with low sensitivity, as just ~200 mRNA reads are acquired from each cell (145). FISSEQ is also relatively slow, as the SOLiD sequencing step requires around 10 days on the microscope.

In spatial transcriptomics (ST), mRNAs are captured from tissue by oligo(dT)-containing probes that have been affixed to a slide in an array of 55-100 µm diameter spots (146). The probes in each spot carry a unique barcode, so that after all captured mRNAs are pooled and sequenced, individual reads can be assigned to their spot of origin. This strategy achieves whole-transcriptome multiplexing at levels of sensitivity comparable to standard next generation RNA sequencing techniques, but at ~10-cell resolutions (146). High-definition spatial transcriptomics (HD-ST) is a variant of ST that achieves significantly higher spatial resolution by affixing barcoded probes to 2 µm diameter beads, which reside in individual wells on a slide (147). HD-ST maintains the whole-transcriptome multiplexing capacity of ST, and operates at resolutions much closer to those of hybridization-based imaging techniques, but the decreased area of tissue that corresponds to each well greatly reduces its RNA capture efficiency and consequently sensitivity (147).

Slide-seq is a similar sequencing-based method that also relies upon barcoded oligo(dT)-containing probes (157). Barcoded probes are affixed to 10 µm diameter beads, which are deposited on a glass coverslip, and SOLiD is used to map each barcode to the corresponding bead (157). The tissue being studied is then transferred onto the coverslip for mRNA hybridization, after which the captured mRNA can be pooled and sequenced (157). The strengths and weaknesses of Slide-seq are similar to those of HD-ST: it achieves whole-transcriptome multiplexing at a moderate resolution, but with relatively low sensitivity (roughly 5% of that of scRNA-seq) (157). Slide-seq has been followed by Slide-seqV2, which replaces SOLiD encoding with a barcoding scheme that is more robust to errors and includes an additional second-strand synthesis step between the reverse transcription of captured mRNA and PCR, making Slide-seqV2 an order of magnitude more sensitive (148).

Successful applications of these RNA-interrogating techniques include investigating intra-patient heterogeneity in the expression of genes associated with neuroendocrine and androgen receptor activity in metastatic prostate cancer (156), characterizing the specific cell subpopulations and phenotypes present at the leading edge of squamous cell carcinomas (158), and identifying three recurring types of spatial cellular communities within PDAC tumours, each with a unique composition of malignant, stromal, and immune cell subtypes (159). Another recent application was the use of Slide-seqV2 to describe transcriptomic alterations induced by neighbouring cells in the immune-suppressive TME that promote tumourigenesis of prostate cancer (160). The authors further combined the spatial data obtained via Slide-seqV2 with computational approaches to ligand-receptor pair identification, which enabled them to identify candidate ligand-receptor pairs that were specifically expressed in neighbouring cells, and hence uncover a specific axis that contributes to prostate TME immunosuppression (160).

## 5 Conclusions

An increasing appreciation for the tremendous inter- and intra-tumor functional and phenotypic heterogeneity that exists in nearly every cancer type, both at the tumour and TME levels, underscores the importance of actualizing the goals of precision medicine for individual cancer patients. Achieving these goals will require expanded access to molecular profiling for more patient tumours and a more complete understanding of what these data mean in the context of spatially heterogeneous TMEs, including how they impact therapeutic response. Historically, the widespread use of mIF and mIHC to interrogate the TME has revealed how the abundance and degree of infiltration of immune and stromal cells impact cancer phenotypes. The application of increasingly sophisticated computational methods to mIF and mIHC data has shown that non-cell autonomous interactions within the TME, driven either through direct cell-cell contacts or indirect mechanisms such as metabolite secretion, greatly influence cellular functions and ultimately tumour behaviour. These advances have already led to the development of promising novel treatment modalities, including immune checkpoint blockade therapy. Recently developed profiling technologies offer the multiplexing capacity and the spatial resolution that are needed to further expand our understanding of tumour biology and therapeutic response. Of these technologies, those that are operable without prohibitive amounts of expertise and that can be made available to many patients by virtue of low cost or high sample throughput hold the most potential for effective translation into the clinic. A combination of increased use of these novel technologies and computational advances that improve the interpretation of multiplexed imaging data will give researchers and clinicians the opportunity to develop and apply treatment protocols tailored to the unique dynamics of every patient’s TME.

## Author contributions

DC: Conceptualization, Visualization, Writing – original draft, Writing – review & editing. AF: Writing – original draft, Writing – review & editing. EM: Conceptualization, Visualization, Writing – original draft, Writing – review & editing. EV: Conceptualization, Writing – original draft, Writing – review & editing. GS: Writing – original draft, Writing – review & editing. KN: Writing – original draft, Writing – review & editing. WWL: Writing – review & editing. CM: Writing – original draft, Writing – review & editing. MG: Writing – original draft, Writing – review & editing. WLL: Conceptualization, Writing – original draft, Writing – review & editing.

## Glossary

**Table:**

APC	antigen-presenting cell
CAF	cancer-associated fibroblast
CODEX	co-detection by indexing
CRC	colorectal cancer
DEI	DNA-Exchange-Imaging
DSP	digital spatial profiling
ECM	extracellular matrix
FISSEQ	fluorescent *in situ* sequencing
HD-ST	high-definition spatial transcriptomics
ICB	immune checkpoint blockade
LPS	lipopolysaccharide
MDSC	myeloid-derived suppressor cell
MIBI	multiplexed ion beam imaging
mIF	multiplex immunofluorescence
mIHC	multiplex immunohistochemistry
NSCLC	non-small cell lung cancer
OSCC	oral squamous cell carcinoma
PDAC	pancreatic ductal adenocarcinoma
RCA	rolling circle amplification
ROI	region of interest
RT	reverse transcription
SABER	signal amplification by exchange reaction
SCFA	short-chain fatty acid
scRNA-seq	single-cell RNA sequencing
SEDAL	sequencing with error-reduction by dynamic annealing and ligation
SOLiD	sequencing by oligonucleotide ligation and detection
ST	spatial transcriptomics
STARmap	spatially-resolved transcript amplicon readout mapping
TAM	tumour-associated macrophage
Tfh	T follicular helper
TLS	tertiary lymphoid structure
TME	tumour microenvironment
TIME	tumour immune microenvironment
TNBC	triple-negative breast cancer
Treg	regulatory T cell.
