# Supplementary material for: Delineating spatial cell-cell interactions in the solid tumour microenvironment through the lens of highly multiplexed imaging
Source: Front Immunol. 2023 Oct 23;14:1275890. doi: 10.3389/fimmu.2023.1275890 (PMC10627006; doi:10.3389/fimmu.2023.1275890)
Supplement: Supplementary file 1 [file Table_1.docx]

**Supplementary Table 1: A non-exhaustive list of well-characterized direct and indirect cell-cell interactions in the tumour microenvironment**

| **Cell Type 1** | **Cell Type 2** | **Type of Cancer** | **Type of Interaction** | **Summary** | **Reference** |
| --- | --- | --- | --- | --- | --- |
| CD8+ | Tumour epithelial cells | Metastatic colon | Direct | Higher percentage of tumour epithelial cells to CD8+ cells linked to increased survival | 32 |
| CD8+ | Tumour epithelial cells | Lung adenocarcinoma | Direct | Recurrence correlated with frequency of CD8+ cells neighbouring tumour epithelial cells | 31 |
| CD8+ CD103+ | Cancer cells | Triple negative breast cancer | Direct | Relapse-free survival linked to high levels of CD8+ CD103+ neighbouring cancer cells | 33 |
| CD8+ | CD3+CD8-FOXP3- helper T cells | Metastatic colon cancer | Direct | Impaired cytotoxic lymphocyte activity, increased Treg activity | 32 |
| CD8+ | B cells | Oropharyngeal squamous cell carcinoma | Direct | Increased proximity led to better prognosis | 34 |
| T cells, B cells and dendritic cells | Within tertiary lymphoid structures | Various | Direct | Presence of TLS linked to improved prognosis; TLS can have superior prognostic value than infiltrating CD8+ cells | 41, 42, 43, 44, 45, 46 |
| F. nucleatum | NK and T cells | Colorectal cancer | Direct | TIGIT signalling leads to downregulation of cytotoxic activity of NK and T cells | 54 |
| F. nucleatum | Cancer cells | Colorectal cancer | Direct | Upregulation of hsa-miR-21-5p to stimulate cancer cell growth and invasion; downregulation of hsa-miR-18a-3p and hsa-miR-4802 to induce autophagy and confer resistance to chemotherapy | 56, 57 |
| PD-L1+ | PD-1+, exhausted CD8+, cancer cells | Various cancers | Direct | Proximity between PD-L1+ and these various cell types have been associated with improved outcomes after checkpoint blockade therapy | 25, 61, 62, 63, 64 |
| CD8+ | PD-L1+ macrophages | Metastatic melanoma | Direct | High expression of PD-L1 in M1 macrophages near CD8+ cells correlated with increased likelihood of response to immune checkpoint blockade | 65 |
| PD-L1+ | CD8+, PD-1+ | HPV+ oral and oropharyngeal cancer | Direct | Frequent interactions correlated with poor outcomes | 66, 67 |
| Treg | CD8+ | HPV- oral squamous cell carcinoma; colorectal cancer with microsatellite instability | Indirect | Proximity linked to poor outcomes | 67, 74 |
| CD4+ and CD8+ | Cancer cells | Non-small cell lung cancer | Indirect | Proximity correlated with poor outcomes | 75, 76 |
| Tregs | CD8+ | Gastric tumours, non-small cell lung cancer | Indirect | Proximity correlated with improved prognosis | 75, 77 |
| Tregs | T cells in tertiary lymphoid structures (TLS) | Breast cancer | Indirect | TLS-localized Tregs correlated with poor patient outcomes | 79 |
| M2 macrophages | Ki67+ cancer cells | Non-small cell lung cancer | Indirect | Proliferation of cancer cells, poor patient survival | 81 |
| M2 macrophages | Cancer cells | Pancreatic ductal adenocarcinoma | Indirect | Higher proximity linked to poor outcomes | 82 |
| M2 macrophages | Cancer cells | Gastric cancer | Indirect | Higher proximity linked to improved outcomes | 83 |
| CD8+ | HLA-DR- (predominantly M2) macrophages | Melanoma | Indirect | Short distances correlated with decreased survival | 85 |
| Cancer cells | Lymphocytes | Various | Indirect | Inhibition of lymphocyte infiltration via kynurenine signalling | 91 |
| Gut microbiome | Cancer cells | Colorectal cancer | Indirect | Secretion of butyrate inhibits cancer cell proliferation and promotes apoptosis | 95, 96, 97 |
| Cancer cells | Various immune cells | Various | Indirect | Secretion of lactate promotes M2 polarization of macropahges and induction of Tregs | 107, 108, 109 |
| Cancer cells | Cancer-associated fibroblasts | Various | Indirect | CAF-cancer cell lactate shuttle in which cancer cells are more proliferative, invasive and migratory | 102, 112, 113, 114, 115 |
| Cancer cells | Stromal cells | Prostate cancer | Indirect | Lactate shuttling (MCT1-MCT4 interaction) correlated with later stage tumors | 116, 117 |
| Cancer cells | Pancreatic stellate cells | Pancreatic ductal adenocarcinoma | Indirect | Secretion of alanine from stellate cells is imported by cancer cells and used for metabolism, furthering tumour growth | 118, 119 |
